# A deformable template method for describing and averaging the anatomical variation of the human nasal cavity

**DOI:** 10.1186/s12880-016-0154-8

**Published:** 2016-10-01

**Authors:** Alireza Nejati, Natalia Kabaliuk, Mark C. Jermy, John E. Cater

**Affiliations:** 1 Department of Engineering Science, University of Auckland, Auckland, New Zealand; 2 Department of Mechanical Engineering, the University of Canterbury, Christchurch, New Zealand

**Keywords:** Human airways, Image registration, Thin-plate splines

## Abstract

**Background:**

Understanding airflow through human airways is of importance in drug delivery and development of assisted breathing methods. In this work, we focus on development of a new method to obtain an averaged upper airway geometry from computed tomography (CT) scans of many individuals. This geometry can be used for air flow simulation. We examine the geometry resulting from a data set consisting of 26 airway scans. The methods used to achieve this include nasal cavity segmentation and a deformable template matching procedure.

**Methods:**

The method uses CT scans of the nasal cavity of individuals to obtain a segmented mesh, and coronal cross-sections of this segmented mesh are taken. The cross-sections are processed to extract the nasal cavity, and then thinned (‘skeletonized’) representations of the airways are computed. A reference template is then deformed such that it lies on this thinned representation. The average of these deformations is used to obtain the average geometry. Our procedure tolerates a wider variety of nasal cavity geometries than earlier methods.

**Results:**

To assess the averaging method, key landmark points on each of the input scans as well as the output average geometry are located and compared with one another, showing good agreement. In addition, the cross-sectional area (CSA) profile of the nasal cavities of the input scans and average geometry are also computed, showing that the CSA of the average model falls within the variation of the population.

**Conclusions:**

The use of a deformable template method for aligning and averaging the nasal cavity provides an improved, detailed geometry that is unavailable without using deformation.

**Electronic supplementary material:**

The online version of this article (doi:10.1186/s12880-016-0154-8) contains supplementary material, which is available to authorized users.

## Background

**Fig. 1:**
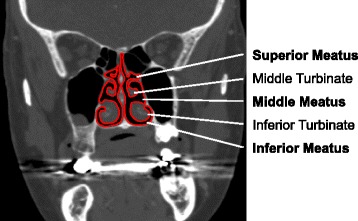
Cross-section of nasal cavity. Cavity outlined in red; meatuses and turbinates indicated. Scan courtesy of the Christchurch Radiology Group (NZ)

Simulation of airflow through human upper airways is of importance in a number of medical applications, including the improvement of artificial respiratory devices [1] for conditions such as obstructive sleep apnea (OSA), and also for delivery of drugs through aerosol deposition [2]. The first step in simulating the flow is producing a geometrical shape. The nasal cavity has two roughly symmetric passages (divided by a thin plate of bone and cartilage called the nasal septum) with each comprised of at least three distinct meatuses (Fig. 1) [3]. Significant variation between individuals is present, such as developmental variations of the underlying bone structure [4, 5] and also the temporal variations that may occur due to the nasal cycle [6]. Deviation of the nasal septum is common [7]. Typically, studies have considered airway shapes obtained from cadaver casts or tomography scans of a single individual [2, 8, 9] or otherwise have studied separately the airflow through the geometries obtained from of a small number (<10) of individuals [7, 10]. They lack universality and may produce quite different airflow results due to the observed variation in nasal cavities of individuals [7].

In a study by Liu et al. [11], nasal cavity geometries from 30 healthy individuals were obtained and used to produce a ‘standardized’ nasal cavity. The procedure involved first segmenting sequences of x-ray computed tomography (CT) scan data from a number of individuals from the transverse plane, then aligning and scaling the individual geometries appropriately, and finally superimposing the geometries and taking mean/medians of each image. This method lacks the ability to work with the various shape deviations that are typically present; for instance it cannot work with deviated nasal septa so only nasal cavities with straight septa were considered. We observe that this does not adequately capture the variations in the population. The method used by Liu et al. also had difficulties coping with congestion (the blockage of the nasal passages due to membranes lining the nose becoming swollen from inflamed blood vessels, which is quite common and difficult to distinguish from tissue/bone in CT scans), and due to the nature of the approach much of the shape information in the superior nasal cavity section was lost.

A somewhat different approach was presented in [12], where the average CT images were used to produce a single geometry. That is, the result is the segmentation of the average scan images, rather than the average of the segmentations of the scan images. However, this approach is also limited to nasal cavities with similar shapes (taken from ethnically uniform females of similar age) and it is not evident how to extend this method to general nasal cavity scans.

**Fig. 2:**
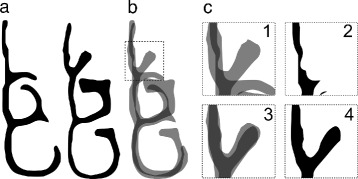
Fourier descriptor limitations. Here, the right nasal cavity section (left side in the image; view from the front) is bounded by a closed curve, which can be represented parametrically as the periodic function *p*, with two points on *p* (*t*=0.5 and *t*=0 which is equal to *t*=1) shown for illustrative purposes. The Fourier decomposition of *p* is thus well-defined (however, an infinite set of parametric functions can be used to represent the boundary, and the choice of *p* is thus to some extent arbitrary). The left section, however, has congestion, thus requiring two closed curves to represent. This complicates the Fourier analysis

In [13, 14], airway shapes were decomposed into a small set of morphological feature coefficients which are then directly averaged. Two feature descriptions were studied: Fourier descriptors and descriptions based on structural decomposition of the medial axis or ‘skeleton’ of coronal nasal cavity cross-sections. The Fourier descriptor method relies on representing finite-length single closed curves as periodic coordinate-valued functions. Applying a low-pass filter on such a function produces a ‘simplified’ shape. There are numerous limitations to applying the Fourier descriptor method. Firstly, for obtaining a mean geometry, a common rotational reference frame must be specified, or else cross-sections must be aligned rotationally. The method also requires modification to be applicable to cross-sections that are not single closed curves i.e. where congestion is present, making a single closed curve insufficient to represent the boundary of the cross-section (see Fig. 2). It is also not known how well the Fourier descriptor method works with shapes that show significant variation.

**Fig. 3:**
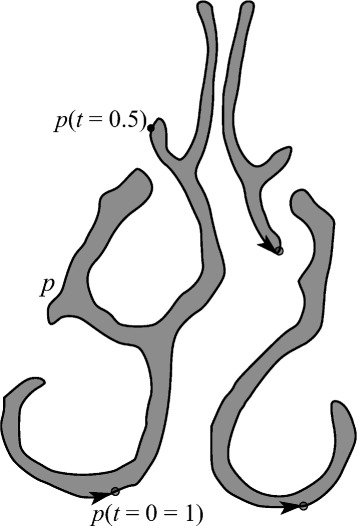
Description of variation in data set. (1) Airway with significant congestion, resulting in filled inferior meatus. (2) Airway with four meatuses. (3) Cross-section where middle and superior meatuses are of small cross-sectional area; (4) larger cross-sectional area, highly pronounced superior meatus, and partially-congested upward-curving supreme meatus. (5) Superimposed images from nasal cavities in [11]. As can be seen, the shape variation is relatively less broad in range

In this work, we study the problem of producing a more representative airway geometry. By this we mean an airway geometry where the size and shape are averaged across airway geometries of a sample of the population. The method presented can accept more shape variation than previously-published methods (see Fig. 3). The contribution of this paper is thus a new shape registration method for the human nasal cavity, with the following advantages:

1. It can succeed with a wider variety of nasal cavity shapes compared to previous methods, including those with different numbers of conchae (3 or 4), deformed septa, congestion, or conchae with differing forms of curvature.
2. It provides a simple way to describe these geometrical variations, allowing for statistical analysis.
3. It is insensitive to variations in nasal passageway cross-sectional area. For instance, during the nasal cycle, or during periods of congestion.

We use a method based on the medial axis description, which consists of finding the ‘skeletons’ or ‘thinned’ representations of airway cross-sections. For instance, a thick vertical slit would be represented by a vertical line passing through the center of the slit. We then use a different type of averaging procedure that is robust and can process geometries with large variations. The method is based on point-set registration [15], in which the resulting skeletons are subdivided into a large set of points and the correspondences between these points and the points from a ‘reference’ or ‘template’ skeleton are discovered.

**Fig. 4:**
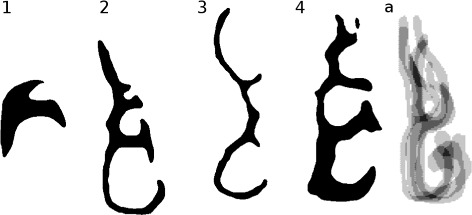
Deformable registration. **a** Two example cross-sections of half of the nasal cavity. **b** The cross-sections are superimposed and aligned. **c**.1 A close-up of the rectangular region in (**b**). **c**.2 Taking the median image results in a mask that conforms poorly with the input shapes. **c**.3 With deformable alignment, we can obtain a better superposition, and **c**.4 a mask that preserves input shapes to a larger degree

The most similar previous work was that of [11]. The primary difference is that no deformable registration was used for alignment of nasal cavities to the templates. We show that the use of deformable registration allows better matching between nasal cavities, and results in an average geometry that includes finer details (see Fig. 4).

## Methods

### Patient CT data sets

Anonymized head and neck CT scans from 26 patients were used to create patient specific airway models. The data set included a mix of male and female (9 male, 17 female) subjects aged from 17 to 70 years, with an average age of 52.7 years and a standard deviation of 13 years. The obtained scans were retrospective and anonymized. No pathologies were observed in the airways. Patients were imaged awake, in a supine position. Scans were comprised of stacks of horizontal slices with 0.43 ×0.43 mm planar resolution; slices were spaced 0.6 mm apart vertically.

### Airway segmentation

**Fig. 5:**
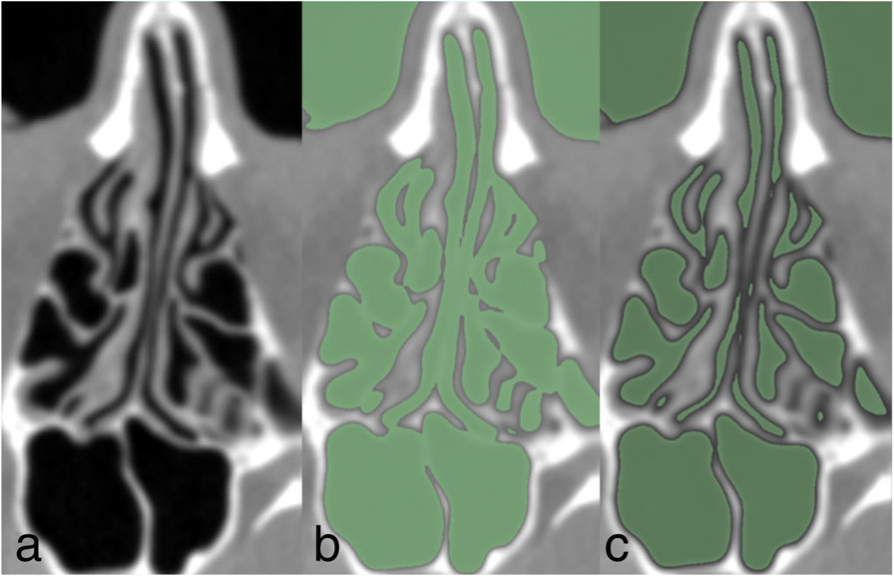
Nasal cavity segmentation. **a** Original, **b** over- and **c** under-thresholded segment of an axial CT image of a nasal cavity and adjacent sinuses. The green colour highlights the segmented area. The reference scale bar length in the lower right corner is 1 cm

The segmentation procedure was as follows: First, the airway from the tip of the nose to the trachea was segmented from other structures in the CT images using 3D Slicer software (v4.3.1) [16]. A thresholding procedure was used for the segmentation of air with the threshold varied from -1000 to -400 Hounsfield units (HU); see Fig. 5. The optimal threshold values varied from scan to scan and within a scan for areas with poor contrast or resolution. The latter was used for the interface between the nasal cavity and paranasal sinuses interface due to the small scale and complex geometry of the nasal cavity passages and sinus drainage openings. Manual slice-by-slice editing was used for these problematic areas to obtain anatomically accurate airway segmentation.

**Fig. 6:**
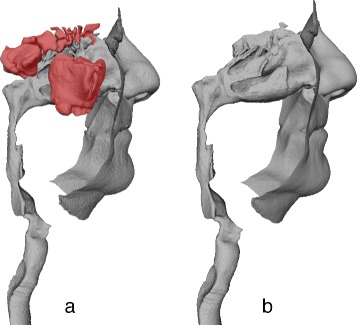
Segmentation post-processing. The 3D surface model of an airway (from the tip of the nose to trachea) **a** before and **b** after sinuses are removed and the model surface is smoothed

In the next step, the segmented image data from all three image planes (axial, coronal and sagittal) was used to create a 3D surface model of the airway using 3D Slicer’s built-in marching cubes algorithm-based ModelMaker module [17]. The procedure was as follows: We applied a default ‘ThresholdEffect’, followed by a ‘PaintEffect’, and then the ‘Unsmooth’ ModelMaker function to generate the surface mesh. The generated model was then exported in an triangular mesh format for editing. MeshLab software (v1.3.3) [18] was then used to remove the sinuses and to smooth the surface of the model (see Fig. 6), using the hole-filling ‘Replace’ module (Smooth mean value coordinates (MVC) method), and the localised robust smoothing, refinement sub-module (default parameters: refinement: 0, reduction: 5.0, smoothing: 5.0). The frontal, maxillary, ethmoidal and sphenoidal sinuses were removed from the nasal cavity by manual editing in MeshMixer software (v10.6.53)^1^. To smooth the airway model surface, while preserving its topology and size, a two-step MeshLab Laplacian Smoothing was applied (1D Boundary Smoothing, cotangent weight method). This procedure resulted in smooth 3D representations of the airway of each patient.

There is a large ‘air’ volume in front of the face which is topologically connected to the air inside the nasal cavity. We remove this volume via a morphological opening of the segmented volume [19], in the anterior volume, by a spherical structuring element of radius *r*. An additional erosion of radius *r*_*ε*_ is used to capture ‘outlier’ areas that spherical structuring elements do not fit e.g. the narrow channel between the nose and the upper lip. The radius *r* must be set to a value that is larger than the internal wall-to-wall dimensions of the nasal cavity but smaller than the volume outside the face. We used a value of *r* = 10 mm.

### 3D mesh model alignment

The absolute coordinates of the origin and relative rotation of each individual varies when CT scans are acquired. The first step in the analysis is the alignment of the airways of scans of different subjects so that they are all approximately in the same spatial position and have the same alignment and scale. This is because we ‘slice’ the scan into cross-sections later, and we require the cross-sections to approximately contain the same features in the structure. For alignment, the common approach is to identify a set of ‘landmarks’ on the shape. Landmarks must be “homologous anatomical loci that provide adequate coverage of the morphology, and can be found repeatedly and reliably” [20]. Specifically, landmarks are zero-dimensional points, and we use the term ‘landmark’ as opposed to ‘feature’ for these points, both to make this distinction and for consistency with the field of geometric morphometrics.

Alignment of meshes according to shape similarity requires specification of seven degrees of freedom corresponding to three translation, three rotation, and one scale degree of freedom [11]. We locate a pair of landmarks in each scan. Bone-based landmarks are preferable to tissue-based landmarks as these are less subject to variation based on experimental conditions and nasal cycle phase. In [11], the following landmarks were selected for this purpose:

- The tip of the anterior maxillary spine (AMS), where the nasal septum and maxilla bone intersect.
- The choana; the beginning of the nasopharynx marked by the point where the two interior nostrils (choanae) meet. The two choanae are the openings between the nasal cavity and the nasopharynx, where the left and right nasal cavity passageways meet.

**Fig. 7:**
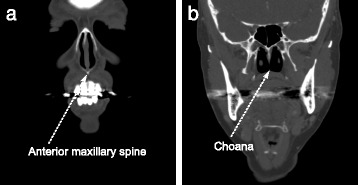
Bone landmarks in CT scans. **a** Maxillary spine landmark **b** Choana landmark

These landmarks are illustrated in Fig. 7 and specify six degrees of freedom. The final degree of freedom is rotation about the line between the landmarks. In [11], an automatic alignment procedure was used to specify this but there is no need to do this for our method since it is insensitive to rotation about this axis. We simply align the airways such that the AMS is at the origin and the line between the two landmarks is parallel to the y-axis (the z-axis is in the upwards direction towards the top of the head, and the x-axis is in the sideways direction). Additionally, once this alignment has been done, the geometry is uniformly scaled such that the choana lies at a distance of 60 mm from the AMS. This is based on the typical average length of the AMS-Choana distance; see, for example [11].

### Anatomical description of the individual nasal cavities

**Fig. 8:**
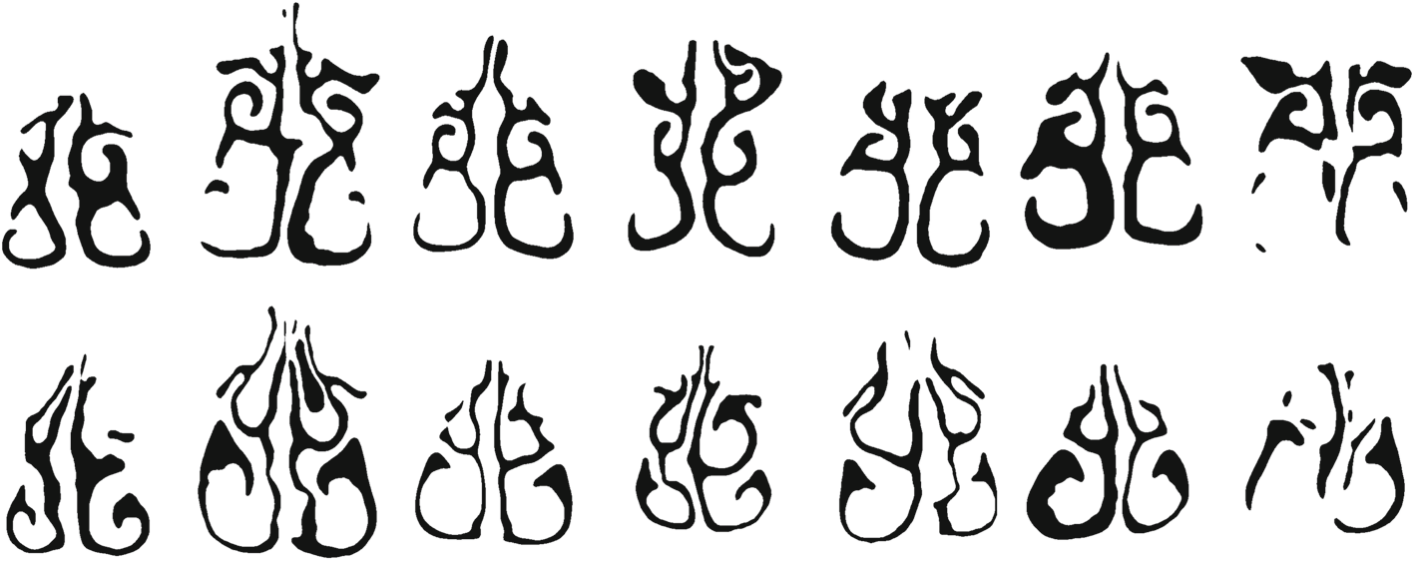
Variation of nasal cavity shapes. Coronal cross-sections of the nasal cavity. *Top row*: Cross-sections approximately 4.5 cm posterior to the AMS landmark. *Bottom row*: Cross-sections taken 3.0 cm posterior to the AMS
- Cross-sectional thickness of the passages due to congestion, the nasal cycle, or inflammation.
- Height of the supreme meatus due to a lack of CT scan data near the orbital plane.
- The curvature of the nasal septum. Variation is common even in healthy individuals [22].

Coronal cross-sections of the nasal cavity are extracted. This converts the 3D geometry to a set of 2D cross-sections. These cross-sections are processed independently. This is a common approach to studying nasal cavity shape since two-dimensional shape analysis techniques are highly developed [11, 12, 21]. For instance, we can robustly identify the inferior and middle sections of the airway [14]. Sections are shown in Fig. 8. The major sources of variability in the airway shapes are:

- Overall dimensions e.g. size of the nasal cavity (which is dependent on the size of the individual as well as other factors).

**Fig. 9:**
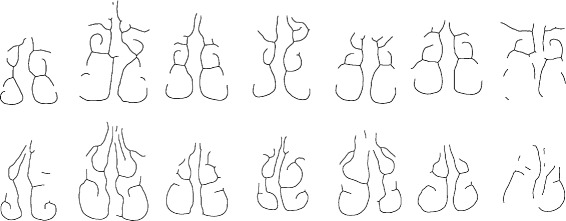
Medial axis transformation. Images correspond to those shown in Fig. 8

We make the observation that, despite the variability in individuals, many airway shapes can be viewed as ‘warped’ or smoothly deformed versions of one another. Such variations are common in biological contexts [23–25]. Even though large variations are present, the various structures (meatuses, etc.) have approximately the same position and alignment with respect to one another. Thus we describe the cross-sections by first obtaining the medial axis (e.g. Fig. 9).

The medial axis of a shape is the set of all points having more than one closest point on the object’s boundary. In practical terms, the medial axis provides a ‘thinning’ or ‘skeleton’ for a shape. Here the medial axis is computed according to the Zhang-Suen thinning algorithm [26], which provides several desirable properties, for example each pixel on an edge has exactly two neighbors, and each pixel at a bifurcation point has exactly three. The branching of the airway into various meatuses can be seen consistently with this medial axis transformation [14].

The use of the medial axis transform has the benefit of eliminating the variability due to the cross-sectional area of the airway. The result of the medial axis transformation is a set of curves. We describe each curve by choosing a set of points that approximate the curve. These points are known as *semilandmarks*. The distinction between semilandmarks and landmarks is that the relative spatial position of landmarks important, but the relative spatial position of semilandmarks is not - we can slide semilandmarks along curves without changing the curve that the semilandmarks define. In other words, if two sets of semilandmarks have different coordinates but define similar curves, we consider the sets of landmarks to be aligned with respect to one another.

Now we envision some ‘reference’ shape template (set of curves) that undergoes a smooth non-intersecting deformation to superimpose on the curves of a desired ‘target’ shape, based on a similarity metric. The deformation process and similarity metric are given in section ‘Matching Procedure’.

In what follows we address the problem of how to deform the reference template to fit target shapes. We desire deformations that are as ‘simple’ as possible in the sense of not requiring complex, convoluted deformations. This is to preserve the spatial relationship of adjacent structures. Also, as we are interested in the position of landmarks, it is desirable to have a deformation formulation that is based on altering the position of (semi)landmarks [20]. That is, we prefer a deformation that can be represented by deforming a small set of ’sample’ points and ’extrapolating’ this deformation to the rest of the space.

To summarize, we require a deformation formulation that has the following features:

- Produces a continuous and smooth deformation that matches the control points
- Invariant to rotation and translation
- Non-affine; this allows us to take into account nasal septum deviations
- Based on landmark positions

Thin-plate splines (TPS) [27] satisfy all of these requirements, and are formulated as follows. Let *x*_*i*_ be a set of control points which are mapped to the points *y*_*i*_. The thin-plate spline is the unique deformation *f* that minimizes the following function,

1 $$ {{} {\begin{aligned} \sum\limits_{i=1}^{K}\|y_{i}-f(x_{i})\|+\lambda \int\int\left[\left(\frac{\partial^{2}f}{\partial x^{2}}\right)^{2}+2\left(\frac{\partial^{2}f}{\partial x\partial y}\right)^{2}+\left(\frac{\partial^{2}f}{\partial y^{2}}\right)^{2}\right]dxdy. \end{aligned}}}  $$

The second term in this equation is the squared curvature of *f*. *λ* is a real-valued stiffness parameter that controls the relative weight of stiffness vs. accurate point matching. This deformation *f* can be found exactly via radial basis functions [28].

### Matching Procedure

**Fig. 10:**
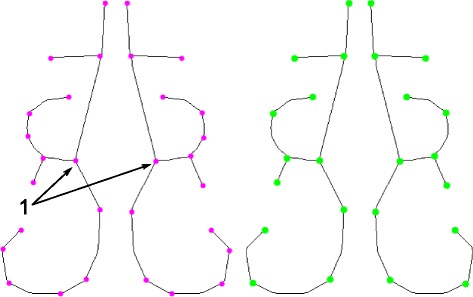
Deformation procedure. **a** Target shape (gray), with skeleton (white) showing semilandmarks (dots). **b** Reference template skeleton (black) showing semilandmarks (dots) and control points (black circles). **c** Reference skeleton fit onto target skeleton; new control point coordinates are stored as the deformation for this target shape

**Fig. 11:**
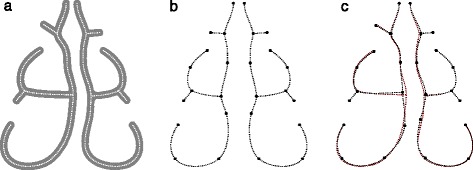
Carleton-Civic geometry. Cross-sectional slices taken from the Carleton-civic geometry, approximately ~1cm apart from the AMS landmark to the choana

The first step of our method is the medial axis transformation, as described. The second step is non-rigid registration of the thinned cross-sections to a ‘template’ consisting of an airway with demarcated sub-regions (using thin-plate splines), using thin-plate splines (see Fig. 10). The template itself is based on the Carleton-Civic airway geometry [11] and is shown in Fig. 11. Even though the Carleton-Civic geometry lacks precision in terms of capturing airway features, for this stage of the process only an approximate skeleton of the airway is required, since we later discard the template’s control point coordinates and use coordinates from the input data.

For finding the optimal deformation, we define the following sets of points:

- The *reference* point set, which in this case is the set of points comprising the medial axis of the Carleton-Civic geometry.
- The *target* point set, which is the set of points comprising the medial axis of an individual airway section.
- The *control points*, which are a small set of points which represent our TPS deformation.

For each pair of slices, we deform the set of control points, use TPS to extrapolate this deformation to the other points, and try to find the deformation that will optimize the alignment of the target and reference points (see Fig. 10).

Let the set of control points in the reference template be {*X*_*i*,*j*_}, where *j* indexes the cross-sectional image number and *i* indexes each control point in a cross-sectional image. After non-rigid registration, we obtain the points {*Y*_*i*,*k*,*j*_} where *k* indexes the target (not reference) cross-section. We compute the average position of each control point $\bar {Y}_{i,j}=\frac {1}{K}\sum _{k=1}^{K}Y_{i,k,l}$ to obtain a new set of control points. We then align all of the cross-sectional images to these coordinates, to obtain a superimposed image where most of the airways overlap. Finally, the median intensity of this image is determined. This completes the description of our algorithm, however two details remain: The pairwise matching algorithm, and the method used for selecting control points. For the pairwise matching procedure mentioned above, we use the method of deformable *robust point matching* (RPM), see [28] for further details.

**Fig. 12:**
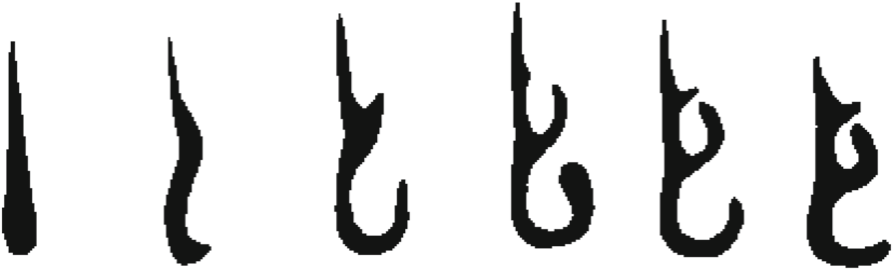
RDP Algorithm. The reference skeleton and example control points produced by the RDP algorithm with *ε* = 1 mm (left) and *ε* = 2 mm (right)

We select control points as follows. There must be enough control points to compactly represent the natural range of deformations that are observed. In theory, if a curve in the reference template can be approximated by a piecewise-linear curve, then the nodes of this curve can be taken as control points and would provide a nearly-complete representation of any possible deformation of the curve that preserves the piecewise-linear structure. Thus, we use the Ramer-Douglas-Puecker (RDP) algorithm [29, 30] on the skeleton to produce a set of control points. The RDP algorithm requires a parameter *ε* representing the characteristic length scale of the resulting approximation. By varying *ε*, the number of control points can be varied, with larger *ε* leading to fewer control points, as shown in Fig. 12.

Our final procedure for obtaining the mean geometry is as follows:

1. Align to landmarks (AMS & Choana)
2. Slice into cross-sections
3. Medial axis transformation on cross-sectional images
4. For each image:

1. Find the thin-plate spline deformation that matches reference skeleton to target skeleton
2. Discard coordinate information of reference control points and use average coordinates of target control points
3. Deform all target images (not skeletons) to average control point coordinates
4. Average all deformed images, filter, and take the median to result in a median image mask
5. Optionally, use the skeleton of the median image mask as a reference skeleton, and repeat steps 1)-4).

**Fig. 13:**
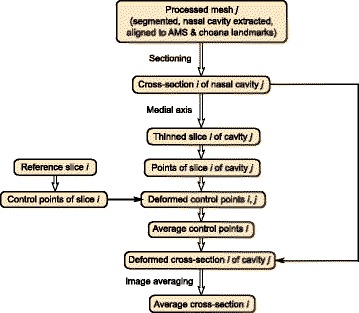
Data flowchart of shape averaging procedure described in text. Boxes represent data; arrows represent operations. Indices *i*=1,…,*N* and *j*=1,…,*N* represent data that consists of arrays of objects; *N* is the number of cross-sections and *M* is the number of individual patient scans (*M*=26, here)

To visualize this procedure, a flowchart is presented in Fig. 13.

### Per-passage CSA calculation

**Fig. 14:**
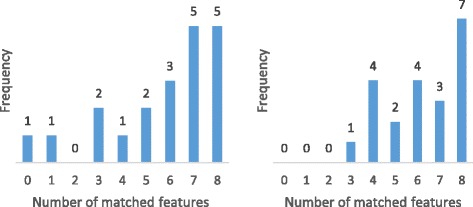
Segmentation of airway components. Distinct passages are identified in different colors based on the location of the middle branch point

We calculate the cross-sectional area (CSA) along the cross-sectional images of the airway, and we also calculate the CSA for the various parts (inferior, middle, superior) of the airway. To do this, the different parts are labelled separately and the number of pixels with each label counted. See Fig. 14.

For labeling the airway sections separately, an automated procedure is used. The traditional anatomical classification of the various sections is ill-defined. For instance, the regions encompassed by the inferior turbinate and main passage overlap. Here, labeling is done based on the skeleton. After localization of the main branch point (the point where the middle turbinate branches from the main passage), the three skeletal branches from this point are cut off at distance *r* = 3 mm (to remove any ambiguity of the skeleton in the neighborhood of the branch point), and then the ‘inferior’ branch is taken to be the branch with most negative minimum in the top-bottom direction. The ‘middle’ branch point is taken to be the branch with the most negative minimum in the left-right direction (assuming the right passageway; for the left passageway this is reversed).

After this, the procedure is to label all other parts of the skeleton. We must allow for ‘broken’ skeletons as well as multiple extraneous branches. This is done via the following process: consider all non-labeled curves in the skeleton, and pick the one with an endpoint closest to any labeled curve in the skeleton. Then label this curve with the same label. This process is repeated until all curves in the skeleton have been labeled.

The final part of the procedure consists of labeling all pixels in the binary mask that are not in the skeleton. Care must be taken here as we cannot simply assign labels based on proximity to nearest curve in the skeleton. This would give incorrect labels in situations where, for instance, a ‘thick’ curve is in close proximity to a ‘thin’ one. The labeling procedure is similar to the one for curves except it is done based on pixels, i.e. at each step we pick the non-labeled pixel that is closest to the set of labeled pixels and we assign this pixel the same label. This process is repeated until all pixels have been labeled. For the purposes of computational efficiency, an optimized nearest-neighbor search is employed (Fast Library for Approximate Nearest Neighbors (FLANN)) [31].

## Geometry results

**Fig. 15:**
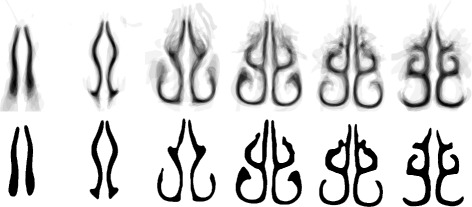
Median slices. *Top*: averaged cross-sectional images, taken at approximately ~1 cm intervals from AMS to choana (left-most image is closest to nose, right-most image is closest to choana). *Bottom*: filtered (*σ* = 0.5 mm) and median-thresholded result

The final result of this procedure is a standardized geometry. This geometry will be referred to as the UoA-UC (University of Auckland - University of Canterbury) standardized nasal model in the figures. The resulting shapes are shown in Fig. 15. The full set of cross-sectional slices is available for download (Additional file 1).

To objectively measure the performance of the deformation procedure, we varied *λ*, the ‘stiffness’ parameter of the deformation procedure. For *ε*, the characteristic length of the RDP algorithm which controls the number of control points, the base value of *ε*=1 was used. After deforming all cross-sectional images, we then calculated how well the images overlapped with each other. We did this using the following metric:

$$\text{Similarity}=\frac{\left|\bigcap_{i=1}^{N}S_{i}\right|}{\left|\bigcup_{i=1}^{N}S_{i}\right|}, $$

where *S*_*i*_ is the deformed image for the scan indexed by *i*.

**Fig. 16:**
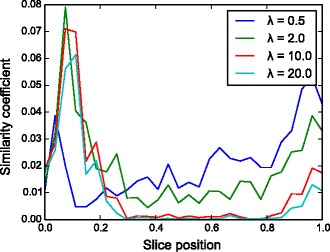
Similarity coefficient. The computed similarity coefficients for different values of *λ* and *ε* = 1 mm. The horizontal axis is the image location which is between the AMS and the Choana

In Fig. 16, from the AMS to the choana, the cross-sectional image shape becomes more complicated and the overlap decreases. Also, the lowest *λ* value performs poorly for the first cross-sections, but performs well for the latter cross-sections. This may be because the anterior cross-sections have fewer control points (being of simpler shape), with better skeletal alignment at the expense of cross-section alignment. Thus different values of *λ* seem to be optimal for different cross-sectional positions. However, the plot illustrates that a very high *λ* value, corresponding to a ’stiff’, non-deforming transformation, is generally suboptimal. This indicates that the method performs better when deformable registration is used.

To measure the performance of the deformation procedure in matching features for each of the individual scans we manually determine if the TPS-based matching procedure has correctly aligned the position of various features of the airway with the template. To do this, we check if the deformed point coordinates are superimposed on the reference point coordinates. For curves, we check if their deformed control points are superimposed on the reference points. The features that were considered are:

- The inferior/middle meatus branch point i.e. the point on each cross-section where the middle meatus and the inferior meatus join (points indicated ’1’ in Fig. 12).
- The curve of the inferior meatus i.e. whether the inferior meatus in the template is properly matched to the inferior meatus in the test slice (black region in Fig. 14).
- The middle meatus curve (green region in Fig. 14).
- The passageway above the middle meatus, including the superior meatus (blue region in Fig. 14).

Each of these four features were considered for both the left and right passageways, resulting in 8 features that could either be properly or improperly matched in each individual scan. We confirmed the proper matching of these features at the control points along the curves; in the nasal valve region the deformation procedure achieved 100 % matching accuracy. For the nasopharynx region, we also found that the outline of the airway is matched in every scan. For the turbinate region, results varied in more posterior sections (no superior meatus) and more anterior sections, thus we show the results separately in Table 1.

**Table 1:** Matching performance of method presented in paper

	Left (a)	Right (a)	Left (b)	Right (b)
Inferior branch point	0.85	0.65	0.81	0.85
Inferior meatus curve	0.85	0.73	0.89	0.77
Middle meatus curve	0.71	0.62	0.77	0.81
Superior curve	0.85	0.69	0.62	0.65

Shown is the proportion of features that are correctly matched using the deformation algorithm. (a) Refers to the area of the nasal cavity where the inferior and middle meatuses are present; (b) refers to the area where the superior meatus is also present

**Fig. 17:**
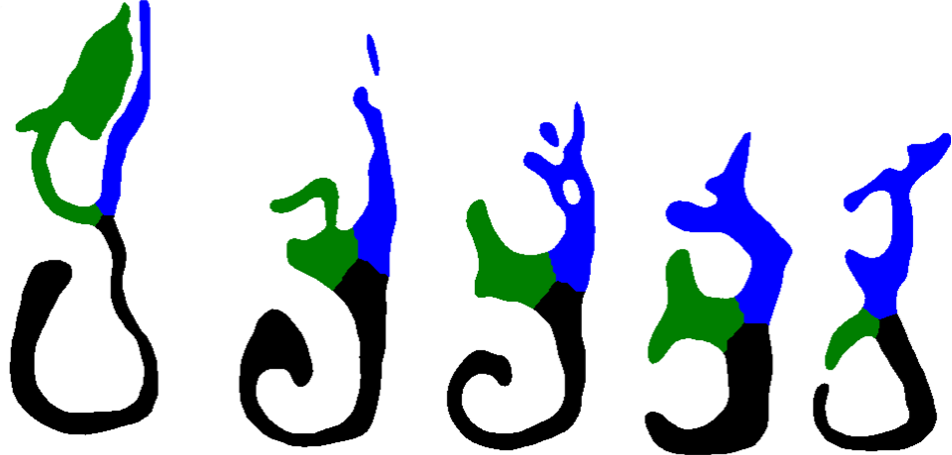
Histogram of per-scan matching performance. *Left*: cross-section 3.5 cm behind AMS; *right*: cross-section 4.0 cm behind AMS (both within turbinate region)

Some scans have less variation and thus match better than other scans. In Fig. 17 we show histograms of matching performance, this time on a per-individual basis. For the majority of individual scans, the method successfully matches all 8 features; however for others some or all of the features fail to properly match. The ‘peak’ near 0 is higher than would be expected if each of the individual features failed to match at a rate independent of the others fail to match any features. This suggests that those features that aren’t matched belong to scans that have outlying variation.

**Fig. 18:**
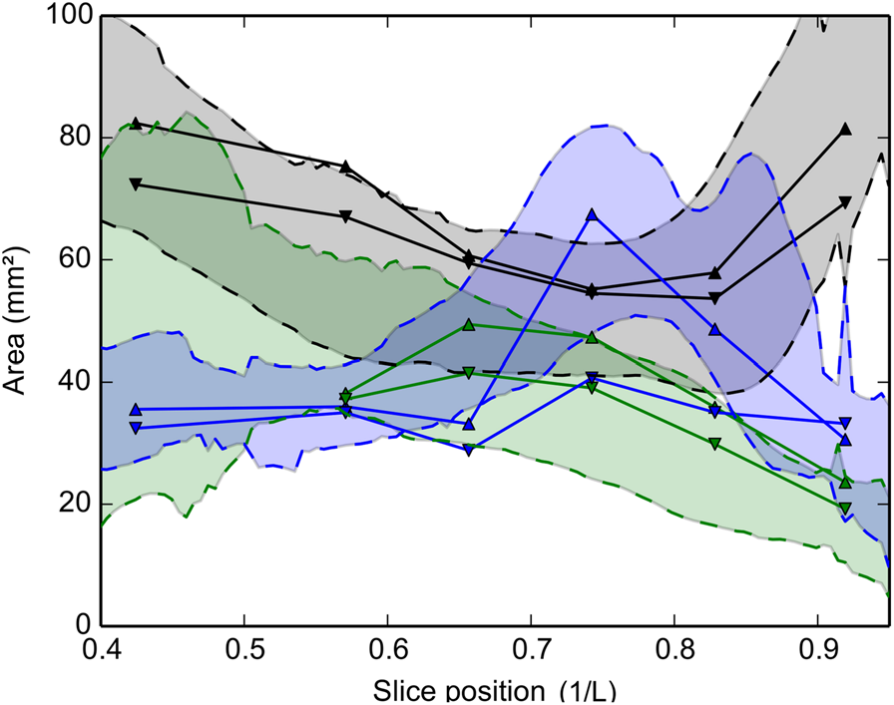
Component-specific cross-sectional areas. Measurements of cross-sectional areas of the left (downward triangles) and right (upward triangles) passageways for the mean geometry (solid line). The variation of measurements of patient geometries are shown as transparent filled areas (*black*=inferior, *green*=middle, *blue*=superior), representing one standard deviation (in both the upward and downward direction) from the mean

To assess the new geometry, we compare the cross-sectional area of each ‘part’ of the geometry (inferior/middle/posterior meatuses) with the individual scans (Fig. 14; procedure detailed in *§*2.5). Each of the separate passageways (inferior, middle, superior), in turn, start at a low value and then gradually peak before decreasing again (Fig. 18). Near the posterior of the cavity, the inferior turbinate section once again increases in cross-sectional area. The measurements from the mean geometry are well within the natural range of variation and, most importantly, reflect the trends of changing cross-sectional area (CSA) at different positions along the cavity.

## Results and discussion

The values of both total CSA and per-section CSA show variation among subjects. A possible reason for this is the nasal cycle. The CSA of the average geometry closely tracks the CSA of the test subjects. In other studies [11, 21] only total CSAs are compared, not per-section CSAs. The mean geometry shows less per-cross-section variation of CSA, especially in the middle meatus section, than the individual subjects. In addition, the mean geometry shows a ‘dip’ in the CSA of the superior meatus near the posterior of the geometry. A possible explanation could be the lack of overlap of the different scans in this section.

**Fig. 19:**
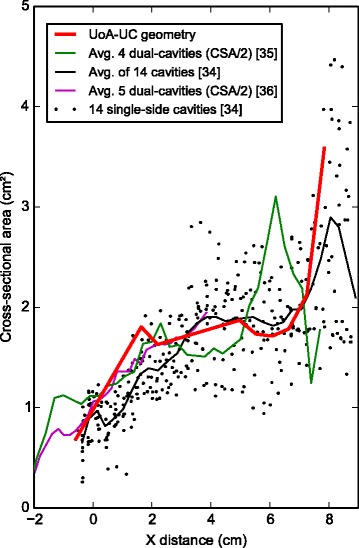
Cross-sectional areas of nasal cavity geometries. Comparison of cross-sectional areas of the UoA-UC geometry (blue curve) with published CT scan and MRI scan derived data from literature. The horizontal axis is distance (in cm) from the anterior tip of the nostril in the coordinate frame used in this study. In the posterior area (near the nostrils) some data sources lack measurements

We also compare the CSAs of the individual sub- jects with CSA results reported in the literature (Fig. 19) [32–34]. It is important to note that due to different alignment systems used in the literature, the CSA values may be offset by some amount. In general, there is less variation in the middle section and the variation increases towards the anterior. An increase in total CSA is apparent towards the anterior. A large increase happens in the nasal valve area (up to 3 cm away from the AMS landmark) and then the CSA remains approximately constant, before a dramatic increase again in the nasopharynx area. CSA variation is more apparent than the rigid nasal cavity as the nasopharynx area is surrounded by more tissue and muscle.

We anticipate the use of the methods outlined in this paper for analysis of differences in nasal cavity geometries in different populations. We expect that our model will lead to more accurate simulations of e.g. assisted breathing using nasal cannulae, drug deposition studies, and also simplify studies of the dynamics of natural breathing, due to the finer precision in reflecting the CSAs of the various sections of the nasal cavity geometries of the population.

## Conclusions

A novel method has been developed for obtaining mean nasal cavity geometries from individual planar CT scans. A skeletonization procedure was used to find the general shape of the airway of each cross-sectional image (and to correct for nasal cycle variations) and then a deformation procedure was used to align all the images to a common reference. The use of deformation is the unique aspect of this method that has not been used in previous studies in the literature. We have compared the similarity coefficient of the method with and without deformation, which shows a marked improvement in overlap of the images when deformation is used. This validates use of deformation for obtaining mean geometries. The geometry developed in this work is of finer resolution and higher quality than other, similar geometries that are currently available.

## Endnote

^1^ Autodesk MeshMixer 3.0. 2015.

## Additional file

Additional file 1 Cross-sections for final UoA-UC geometry. File is a HDF5 data set containing cross-sectional slices for computed mean geometry. (H5 347kb)

## Abbreviations

AMS: Anterior maxillary spine
CSA: Cross-sectional area
CT: Computed Tomography
HU: Hounsfield units
MRI: (Nuclear) Magnetic resonance imaging
MVC: Mean value coordinates
OSA: Obstructive sleep apnea
RDP: Ramer-Douglas-Puecker algorithm
RPM: Robust point matching
TPS: Thin-plate spline
